# Milk fermentation by monocultures or co-cultures of *Streptococcus thermophilus* strains

**DOI:** 10.3389/fbioe.2022.1097013

**Published:** 2022-12-12

**Authors:** Mei Han, Yanfeng Wu, Xiaojuan Guo, Lili Jiang, Xin Wang, Zhonghui Gai

**Affiliations:** ^1^ Shanghai Business School, Shanghai, China; ^2^ Department of Research and Development, Wecare Probiotics Co., Ltd., Suzhou, China; ^3^ Instrumental Analysis Center, Shanghai Jiao Tong University, Shanghai, China

**Keywords:** *Streptococcus thermophilus*, fermented milk, acidification rate, post-acidification, viscosity

## Abstract

Direct vat-set starter cultures are the key ingredient for the production of fermented dairy products. The characteristics of the strains used for fermentation determine the fermentation time, texture and flavor of the fermented milk products. In this study, a large-scale analysis of the acid production rate, texture, carbon source utilization characteristics of *Streptococcus thermophilus* strains was conducted. All 100 *S. thermophilus* strains were divided into six groups according to the acid production rate and into two groups according to the consistency texture. A universal medium, basing on the carbon sources metabolic properties were optimized (0.5% lactose and 3.5% glucose), to culture all of the tested strains. Among them 40 strains were used to test pH-controlled conditions using this universal culture medium. After 5–7 h of fermentation, the optical density (OD) values of all fermented products exceeded 10, suggesting the potential for high-density cultivation of *S. thermophilus*. Although the OD could be further increased by adding more glucose, this may have hindered subsequent lyophilization because of high residual lactic acid in the fermented product. Next, the application of *Streptococcus thermophilus* strains in fermented milk was studied. Monocultures and co-cultures of strains were evaluated and compared. The results revealed the existence of symbiotic or competitive relationships between different *S. thermophilus* strains. Based on the findings, the mixing ratio of three symbiotic *S. thermophilus* strains was optimized. A co-culture of these three strains yielded fermented milk with high viscosity, low post-acidification, good sensory properties and processability.

## Introduction

Yogurt is one of the most popular fermented milk products worldwide. Its texture depends on various factors, especially the milk composition, the manufacturing process, and the starter culture used for fermentation (Nguyen et al., 2018; Tarrah et al., 2018). *Streptococcus thermophilus* is a predominant lactic acid bacterium (LAB) with rapid acidification capability. It is a major dairy starter bacterium used to produce yogurt (Liu et al., 2019; Yu et al., 2020). The acidification rate and extracellular polysaccharides (EPS) production by *S. thermophilus* influences the gel formation and final texture of the fermented product (Pachekrepapol et al., 2017). The major bacterial species used for yogurt production include *S. thermophilus* and *Lactobacillus bulgaricus.* During the fermentation process, *S. thermophilus* grows rapidly and produces organic acids that stimulate the growth of *L. bulgaricus* (Yamamoto et al., 2021; Yu et al., 2021). The high acidification rate shortens the fermentation duration, and is beneficial for rapid yogurt production in the dairy industry.

*S. thermophilus* strains from diverse sources have different phenotypic traits. Some studies have defined a growth score and identified a missense mutation (Zhao et al., 2021) or the existence of cell-envelope proteinase- (*PrtS*) (Dandoy et al., 2011; Boulay et al., 2020) and urease-encoding genes (Zotta et al., 2008; Arioli et al., 2017; Yamauchi et al., 2019) to predict the relationship between the genotype and fermentation characteristics of the strains. One disadvantage of these methods is that they can only be applied to investigate bacterial monocultures. In industrial applications, commercial starter cultures for yogurt fermentation usually consist of several strains of *S. thermophilus*, which are chosen for their specific properties such as fast acidification, texture-promoting capacity, and phage resistance ability (Mostafaie et al., 2018; Nguyen et al., 2019). Although the phenotypic characterization of *S. thermophilus* strains would be an extensive and time-consuming task, this method is reliable and repeatable and can reflect the symbiotic characteristics of different strains.

In this study, 100 *S. thermophilus* strains were isolated from naturally fermented dairy products procured from different regions of China, and their fermentation characteristics were explored. In addition, a universal culture medium for high-density cultivation of all the *S. thermophilus* strains was developed. Finally, the effects of monocultures or co-cultures of different *S. thermophilus* strains on the rate of acidification, post-acidification, and the viscosity of fermented milk were studied. The findings of this study lay the foundation for the application of *S. thermophilus* strains with different phenotypic features for yogurt production.

## Materials and methods

### Bacterial strains and growth conditions

One hundred *S. thermophilus* strains were isolated from 80 naturally fermented dairy products, including koumiss, kurut, fermented cow milk, and fermented goat milk, from six provinces in China. These strains were identified by 16 S rDNA sequencing and stored in Wecare Probiotics Co., Ltd.

The isolated *S. thermophilus* strains were cultured in M17 broth (Oxoid, Basingstoke, United Kingdom) supplemented with 1% lactose at 37°C for 16–18 h in an anerobic environment. Their optical density (OD) was tested after 5 times diluted with distilled water at 600 nm. The bacterial cells were collected by centrifugation at 7,000 × *g* for 5 min, and the collected cells were washed once with sterile saline and resuspended in saline for further use. Monocultures were prepared by inoculating at a concentration of 1 × 10^6^ cfu/mL *S. thermophilus* strains individually in fresh milk pasteurized at 95°C for 5 min (Brightdairy, Shanghai, China) or UHT milk. The co-cultures of different *S. thermophilus* strains were prepared by inoculating at a ratio of 1:1 in fresh milk pasteurized at 95°C for 5 min at a total concentration of 1 × 10^6^ CFU/mL. For milk fermentation the temperature was maintained at 42°C.

### Determination of the acidification profile

Acid production during fermentation was expressed as changes in pH and increases in the titratable acidity (TA) of the final product. After fermentation, the fermented yogurt samples were titrated with 0.1 mol/L NaOH (Merck, Darmstadt, Germany) under continuous magnetic stirring until pH 8.30 was reached. The amount of 0.1 mol/L NaOH (mL) required to titrate 100 g of yogurt was referred to as the TA. The pH changes of the samples were determined from the start of the fermentation every 5 min using a Cinac pH meter system (Alliance Instruments, AMS Company, France).

### Optimization of carbon sources

A fermentation medium containing different carbon sources (initial concentration of 40 g/L), yeast extract (10 g/L), peptone (10 g/L), sodium acetate (2 g/L), sodium glutamate (2 g/L), K_2_HPO_4_ (2 g/L), Tween 80 (1 g/L), MgSO_4_ (0.25 g/L), and MnSO_4_ (0.05 g/L) was prepared. All of the ingredients used to prepare the medium were purchased from Sinopharm (Beijing, China). The initial pH was adjusted to 6.8 ± 0.2, and the medium was sterilized at 121°C for 20 min.

pH-controlled fermentation by each strain was carried out in a 3-L jar fermentor (Korea Fermentor Co., Inchon, Korea) containing 2 L medium with an inoculum size of 2% (v/v). All experiments were performed in triplicate.

### Freeze-drying

Cells were harvested under aseptic conditions at the beginning of the stationary phase of fermentation by centrifugation at 10,000 × *g* and 4°C for 15 min (Eppendorf Company, Hamburg, Germany). The growth medium was decanted, and the harvested cells were re-suspended in the cryoprotective medium containing glycerol, sodium glutamate, and skim milk, as the protective agents, suspended in distilled water to obtain cell suspensions containing approximately 1.0 × 10^10^ CFU/mL. The samples were then freeze-dried (FreeZone 6L, Labconco, America).

### Determination of the viscosity profile

When the TA of the yogurt samples reached 70°T, the samples were stirred to stop fermentation and stored in a refrigerator (4°C) for 24 h before testing. The viscosity of all the yogurt samples was then tested using a rotary viscometer (proRheo, R180, Germany) at a rotational speed of 64/s and an interval time of 10 s.

### Human sensory evaluation

A sensory analysis was conducted to evaluate the sensory properties of the fermented milk products. Twenty trained panelists were invited and asked to score the consistency, stringiness, smooth, sweetness, and aroma of the samples using a 9-point intensity scale (1 = *disliked extremely*; 9 = *liked extremely*). The panelists were guided to taste the fermented samples and drink water between the samples to cleanse the palate. Scores were calculated as means of twenty trained panelists.

### Rheological properties of the fermented samples

The rheological tests of the yogurt samples were performed using an ARES-G2 rheometer (TA Instruments, United States) with parallel titanium plate geometry (50 mm diameter, 1 mm gap). The exposed surfaces of the samples were covered with a thin layer of dimethylsiloxane to avoid dehydration. A thixotropic loop test was conducted by increasing the shear rate from 0.1/s to 100/s and then decreasing the shear rate at the same rate, and do this again. The storage modulus (G′) and loss modulus (G″) of the samples were measured by oscillation tests. A stress sweep test was performed to determine the range of linear viscoelasticity. Thereafter, frequency sweeps ranging from 0.1 to 100 rad/s were implemented within the linear viscoelastic region (γ = 0.5%) to obtain the relationships with G′ and G’’. All of the rheological measurements were performed in duplicate.

## Results and discussion

### Acid-producing ability of *S. thermophilus*

**FIGURE 1:**
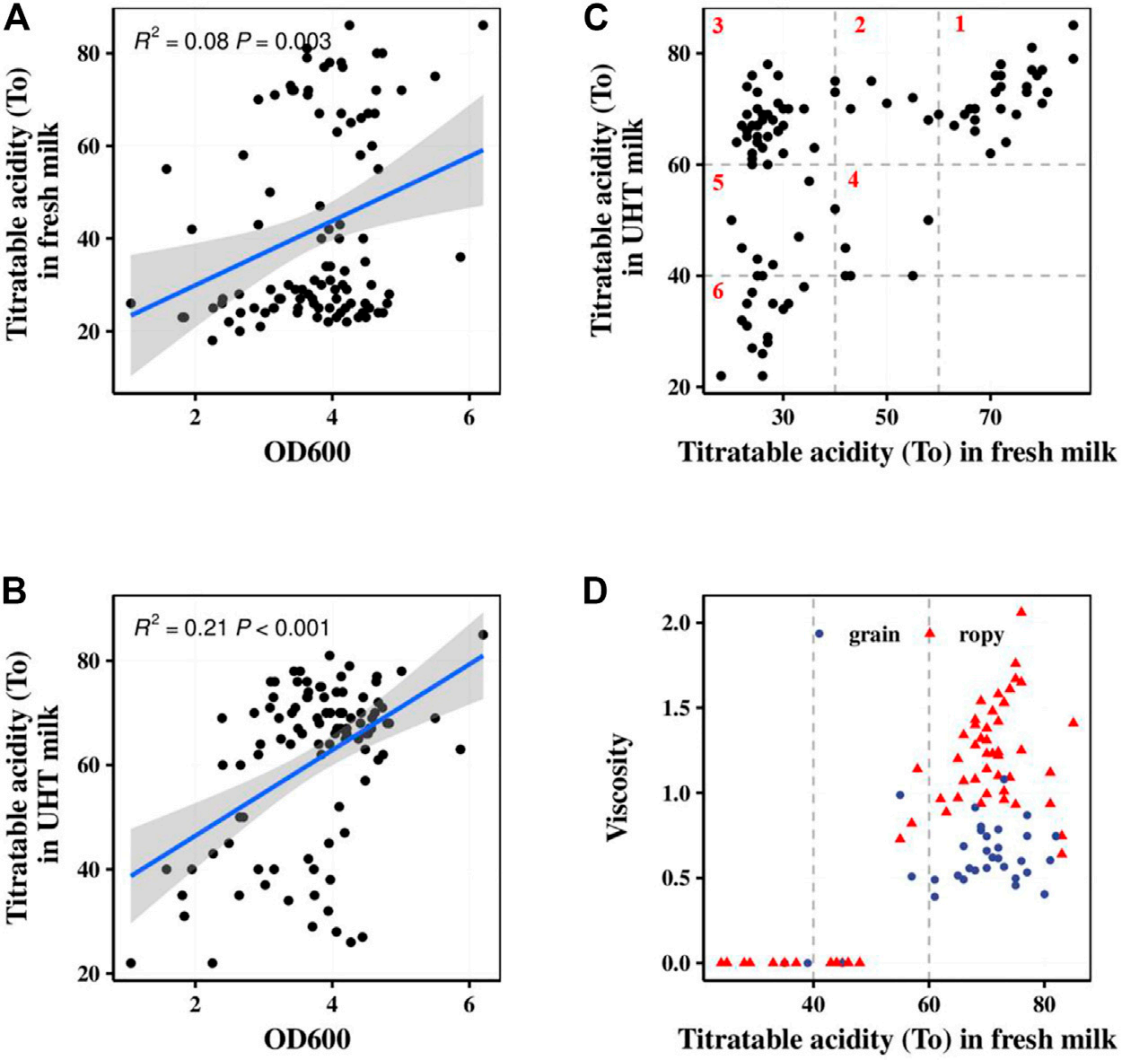
Fermentation characteristics of 100 *S. thermophilus* strains. **(A)** Correlation analysis between lactic acid production in fresh milk (fermentation time 6 h) and OD. **(B)** Correlation analysis between lactic acid production in UHT milk (fermentation time 5 h) and OD. **(C)** Classification of 100 *S. thermophilus* strains according to their acid production characteristics in fresh milk (fermentation time 6 h) and UHT milk (fermentation time 5 h). **(D)** The viscosity and texture of 100 *S. thermophilus* strains in fresh milk for 8 h fermentation.

The acid- and EPS-producing abilities of 100 *S. thermophilus* strains were studied after 5–6 h of fermentation. The role of *S. thermophilus* in starter cultures is related primarily to its fast growth and acid-producing capability (Vendramin et al., 2017). Their lactic acid production ability was positively correlated with their growth ability (Fig. 1AB). According to the acidification rates of different strains in pasteurized fresh milk and UHT milk, all of the strains were divided into six major categories as shown in Figure 1C. Class 1 contained 27 strains with the best acid-producing capability. These strains could grow independently in pasteurized fresh milk, and their acidification rates were stable in different types of milk. Strains in Classes 2 and 3 could rapidly produce acid, but their acidification rates were depended on the milk sources. Strains from Classes 4–6 could not be used alone, but rather only as assistant strains to improve the flavor or texture of the fermented products. Zhao et al. (2021) evaluated the acidification capability of 85 strains of *S. thermophilus* based on the TA and the acid production rate. The TA achieved with and the acid production rate of strains carrying the *PrtS* gene were significantly higher than those of strains not carrying this gene, and these strains were clustered together in the phylogenetic tree.

Of all the tested strains, 34% could not produce enough EPS to improve the texture of the fermented milk as shown in Figure 1D. The texture of their fermented samples was rough, and the graininess and lumpiness of large protein aggregates was usually present in their fermented samples. The other 64% strains generated enough EPS to produce smooth and thick fermented samples in which no or very few small grains (1–2 mm) were observed. These results are in agreement with those reported by Nguyen et al., (2018) and Hassan et al., (2003), who observed reduced graininess in yogurt fermented with a high EPS-producing culture compared with low-EPS producing cultures (Hassan et al., 2003; Nguyen et al., 2018).

### Optimization of culture conditions for *S. thermophilus*

Before *S. thermophilus* strains can be used for milk fermentation in the industry, they need to be cultured in a suitable medium under optimal conditions to increase their growth to yield high cell density. After such culturing followed by centrifugation, bacterial suspensions containing large amounts of cells are used to produce directed vat-set (DVS) starter cultures with which to ferment milk.

Several types of strains are used to produce different DVS starter cultures (de Vin et al., 2005; Settachaimongkon et al., 2014; Karadeniz et al., 2021), so the composition of the culture medium needs to be changed according to the different strains used to prepare the DVS starter culture. In our study, we found that the carbon source was the primary limiting factor for preparing a universal culture medium for different *S. thermophilus* strains.

**FIGURE 2:**
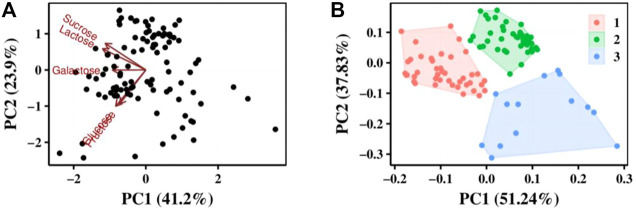
Analysis of 100 *S. thermophilus* strains by carbon sources use ability. **(A)** PCA analysis results, the value of coordinate axis PC1/2 is the explanation rate of the overall difference, the arrow represents the original variable, where the direction represents the correlation between the original variable and the principal component, and the length represents the contribution of the original data to the principal component. **(B)** Strains were divided into three groups according to their utilization of carbon sources by cluster analysis.

Carbon source utilization capacity of the strains tested in our study is shown in Figure 2. When using glucose as the carbon source, 36 strains could not grow, with the OD_600_ being <0.1 after 6 h of fermentation by these strains at 37°C. Similarly, five strains could not grow when using sucrose as the carbon source, only seven strains showed a weak growth when using fructose as the carbon source, and none of the strains showed growth when galactose was used as the carbon source.

Principal component analysis (PCA) was performed using prcomp function for carbon source utilization in different strains, and the effect of carbon source on principal components was investigated (Figure 2A). Cluster analysis was performed using kmeans function to classify the utilization of different carbon sources by strains based on PCA analysis result (Figure 2B). All the strains can be divided into 3 clusters. The first cluster has 42 strains and they can grow well in glucose, sucrose and lactose. The second cluster has 44 strains and they grow weak in glucose. The third cluster has 14 strains they all grow weak both in glucose and sucrose. This revealed that lactose was the best carbon source for all *S. thermophilic* strains.

*S. thermophilus* is a homofermentative bacterium that ferments lactose *via* the Embden–Meyerhof pathway (EMP) to lactate. When *S. thermophilus* is cultured in milk, lactose is transported into the bacterial cell in association with the expulsion of galactose *via* an antiport system. Lactose is then hydrolyzed by bacterial β-galactosidase, but only glucose is metabolized further *via* the EMP to lactate. Overall, 1 mol of lactose is fermented to 2 mol of lactate plus 1 mol of galactose. Most of the *S. thermophilus* strains are galactose-negative, that is, they are unable to efficiently metabolize galactose. This metabolic defect is due, in part, to the production of insufficient levels of galactokinase, a key enzyme of the Leloir pathway encoded by *galK* (Vaillancourt et al., 2002; de Vin et al., 2005). In the fermentation course using lactose as the carbon source is not economical not only because lactose costs approximately three times more than glucose but also the galactose produced from lactose metabolism remains unused. As reported by Filip de Vin et al., 2005 only 8 of the 49 *S. thermophilic* strains they tested could completely utilize lactose, while the other 41 strains could process little or none of the secreted galactose.

Thus, a composite carbon source (0.5% lactose and 3.5% glucose) was used, instead of pure lactose, to prepare the universal culture medium in our study. We found that 0.5% lactose helped to begin fermentation by all glucose-non-fermenting strains, after which 3.5% glucose helped maintain the rapid growth of all strains. After optimizing the carbon source, all 100 strains of *S. thermophilic* were successfully cultured under the same fermentation conditions. This suggests that our optimized culture medium is conducive to large-scale fermentation using different *S. thermophilic* strains.

### Fed-batch culture of *S. thermophilus*

**FIGURE 3:**
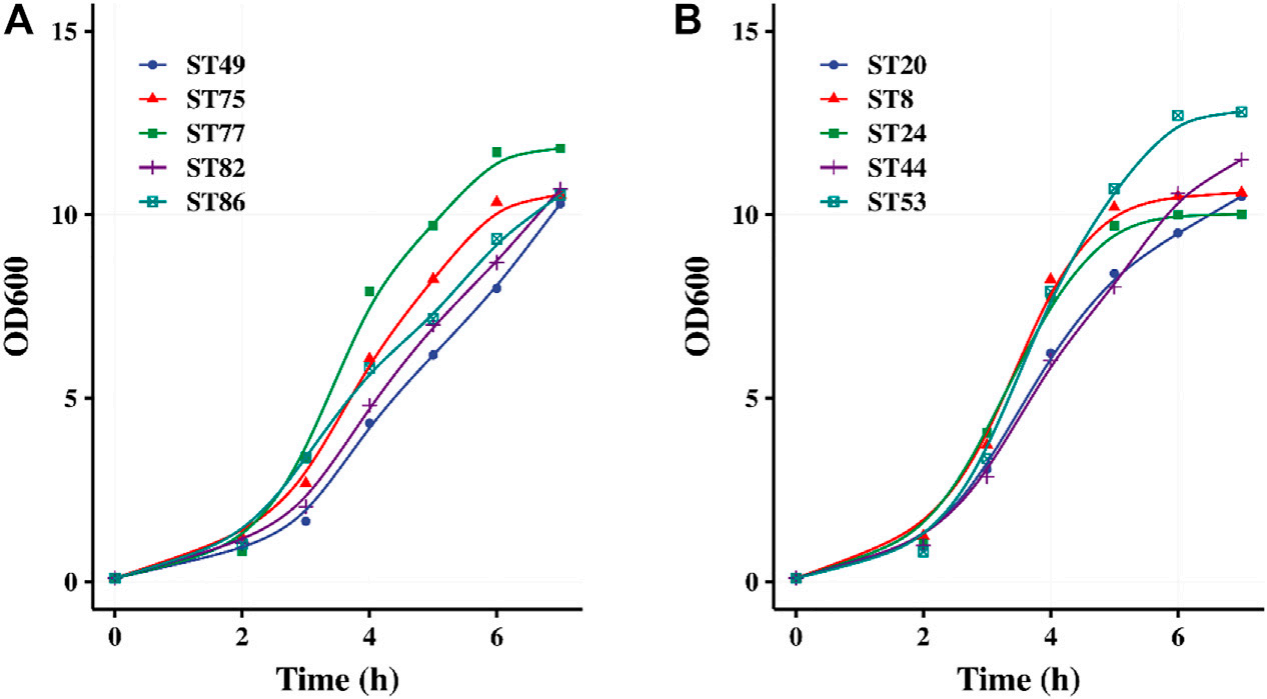
pH controlled fermentation of 5 glucose-non-fermented **(A)** and 5 glucose-fermented **(B)** *S. thermophilus* strains.

Forty strains were used for pH-controlled fermentation using 0.5% lactose and 3.5% glucose as the carbon source. After 5–7 h of fermentation, the OD_600_ values of their fermented products all exceeded 10, indicating that high-density cultivation was achieved. The growth curves of five glucose-non-fermenting strains and five glucose-fermenting strains are shown in Figures 3A,B, respectively. After 2 h of fermentation, all of the 40 strains entered the logarithmic growth phase. Lactose promoted the rapid initiation of fermentation by the glucose-non-fermenting strains, after which these strains continued utilizing glucose similar to the glucose-fermenting strains.

**FIGURE 4:**
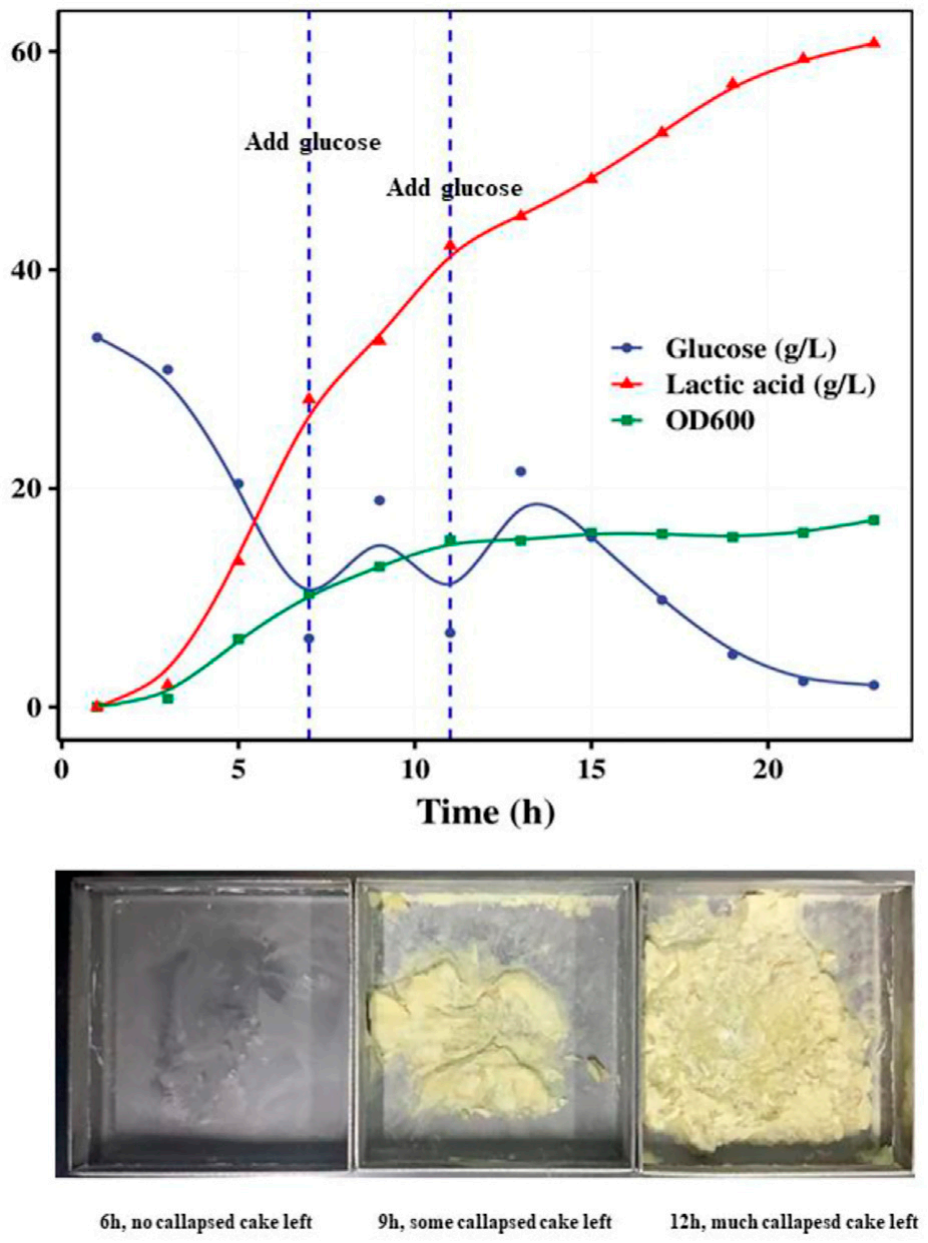
Fed-batch fermentation of strain ST20 and callapsed cakes left in drying trays in different fermentation time.

Glucose fed-batch fermentation was also studied to increase the growth of *S. thermophilus*. The growth curve of strain ST20 under pH-controlled fermentation with glucose supplement was shown in Figure 4. When glucose was supplied during the course of fermentation, the OD_600_ continued increasing until an OD_600_ of 15.8 was reached after 12 h of fermentation, while the lactic acid content continued increasing until all of the supplemented glucose was consumed. The lactic acid in the system reached 60 g/L after 20 h of fermentation.

The cell cultures were collected by centrifugation of fermented samples obtained at 6, and 12 h and mixed with the same weight of cryoprotective solution separately. Each of these prepared bacterial suspensions was then freeze-dried and stored at −70°C. We found that the high levels of lactic acid in the fermentation medium affected the freeze-drying process of the products and even led to the failure of freeze-drying because the low Tg (−60°C) of lactic acid decreased the melting point of the suspension. Particularly, we found that the 9-h and 12-h suspensions had collapsed powder cakes stuck to the drying trays after freeze-drying. For culture broth samples containing more than 35 g/L of lactic acid, we recommend that the concentrated cell suspensions obtained after centrifugation be rinsed with buffer to remove lactic acid before freeze-drying.

### Compound application of different strains

The main roles of the starter culture used for yogurt production are acidification through the conversion of lactose to lactic acid, creation of viscous texture by denaturation of proteins and production of exopolysaccharides, and development of the typical yogurt flavor. After termination of fermentation, the yogurt is stored until consumed. During this period, the LAB may cause further acidification of the product, particularly when stored at temperatures above 4°C. This phenomenon is called post-acidification and is generally considered undesirable, as it may contribute to defects such as syneresis, reduction in viable LAB counts, accumulation of lactic acid in the product, and development of an undesirable flavor (Han et al., 2012; Wang et al., 2013; Mani-López et al., 2014; Akgun et al., 2018). Thus, starter cultures with a mixture of strains capable of rapid acidification, low post-acidification, and creating a viscous texture are favorable. Han et al. showed that the use of Cu^2+^ at the concentration of 1.25 mg/kg (CuSO_4_ 3.15 mg/kg) could reduce post-acidification (Han et al., 2012), but the concentration of this supplemented CuSO_4_ was too high compared with the DVS starter culture usually added at 30 mg/kg.

**FIGURE 5:**
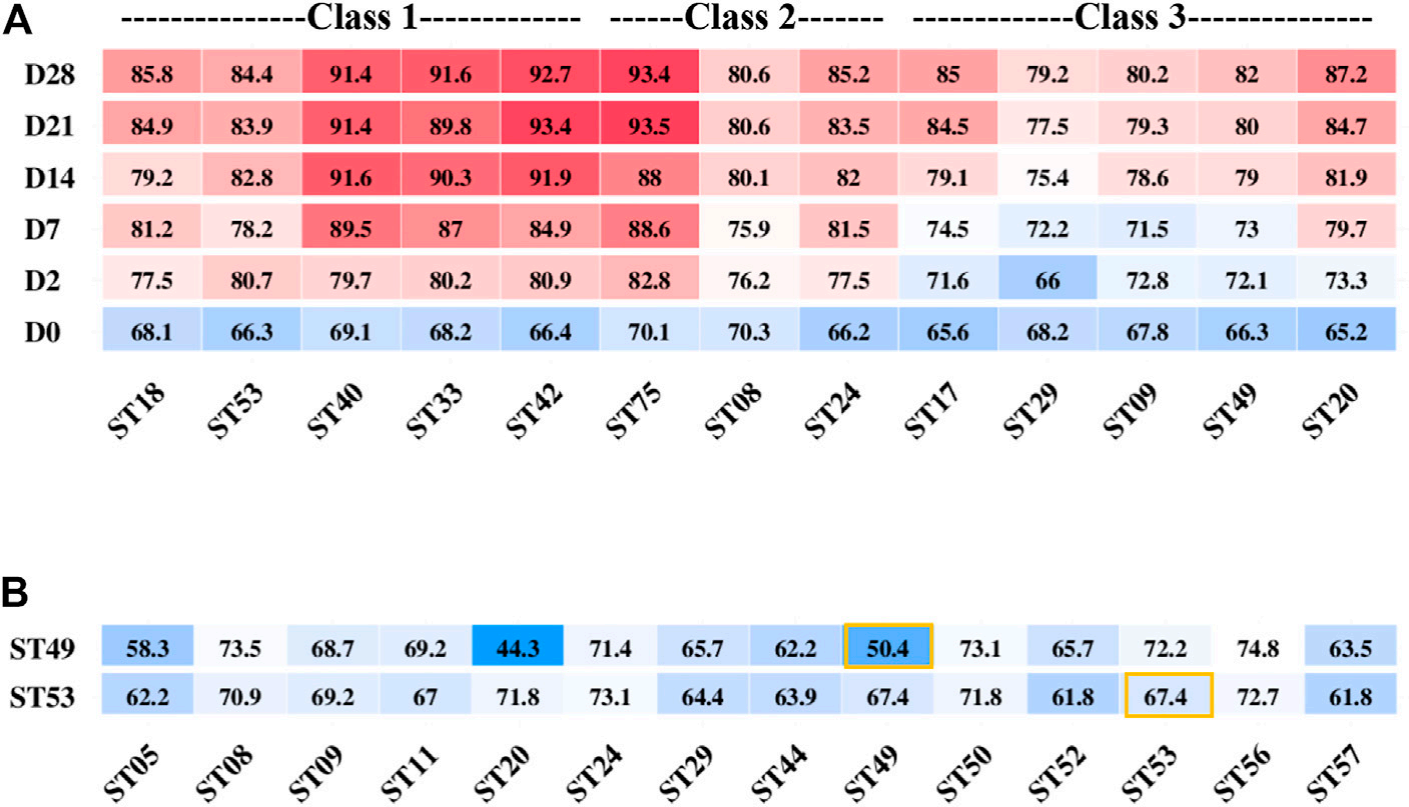
Post-acidification of different *S. thermophilus* strains after the titration acidity reached 70°T **(A)** and titration acidity of ST49 and ST53 fermentation mixed with other strains for 6 h **(B)**.

Class 1–3 strains (Figure 1C) had the ability to produce enough acid for fermentation. The post-acidification capacity of these strains was investigated by testing their fermented products stored at 10°C for 28 days after the TA reached 65–70°T. The strains ST40, ST33, ST42, and ST75 showed the highest post-acidification capability. Notably, although among all classes, Class 1 strains showed the highest post-acidification ability (Figure 5A), we found that the post-acidification ability was related to the strains’ acid fermentation rates that were used to classify the groups.

Next, we used combinations of different strains to improve the yogurt fermentation rate and texture and control post-acidification. Two strains ST53 and ST49 belonged to different classes that showed low post-acidification were selected to study their growth characteristics and interactions when used together with other strains for fermentation.

ST49 was found to be a high-EPS-producing strain but with low proteolytic capacity. When ST49 was co-cultured with other 13 EPS-producing strains at a ratio of 1:1, all of the pair-wise strain combinations except with ST20 increased the fermentation rate (Figure 5B). These results suggest that different *S. thermophilus* strains may promote or inhibit one another’s growth.

The acid production rate of ST53 was high, but there was no accumulation of EPS, so the texture of the product was rough when used ST53 alone for fermentation. Co-cultured with other 13 EPS-producing strains at a ratio of 1:1, we found that ST53 combinations with ST20, ST50, ST56, and ST49 produced smooth-textured yogurt after 6 h of fermentation, whereas its combinations with ST44, ST29, ST05, ST52, and ST57 significantly decreased the fermentation rate (Figure 5B).

**FIGURE 6:**
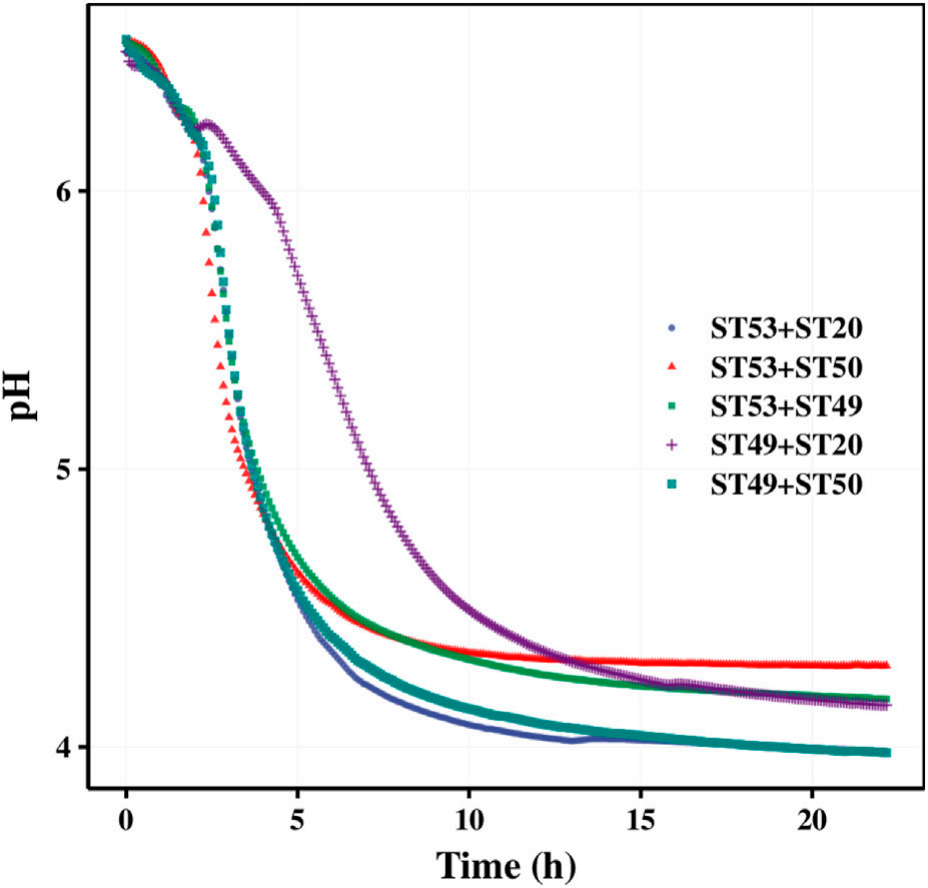
Acid production curve of two strains (Mean of 3 measurements).

**FIGURE 7:**
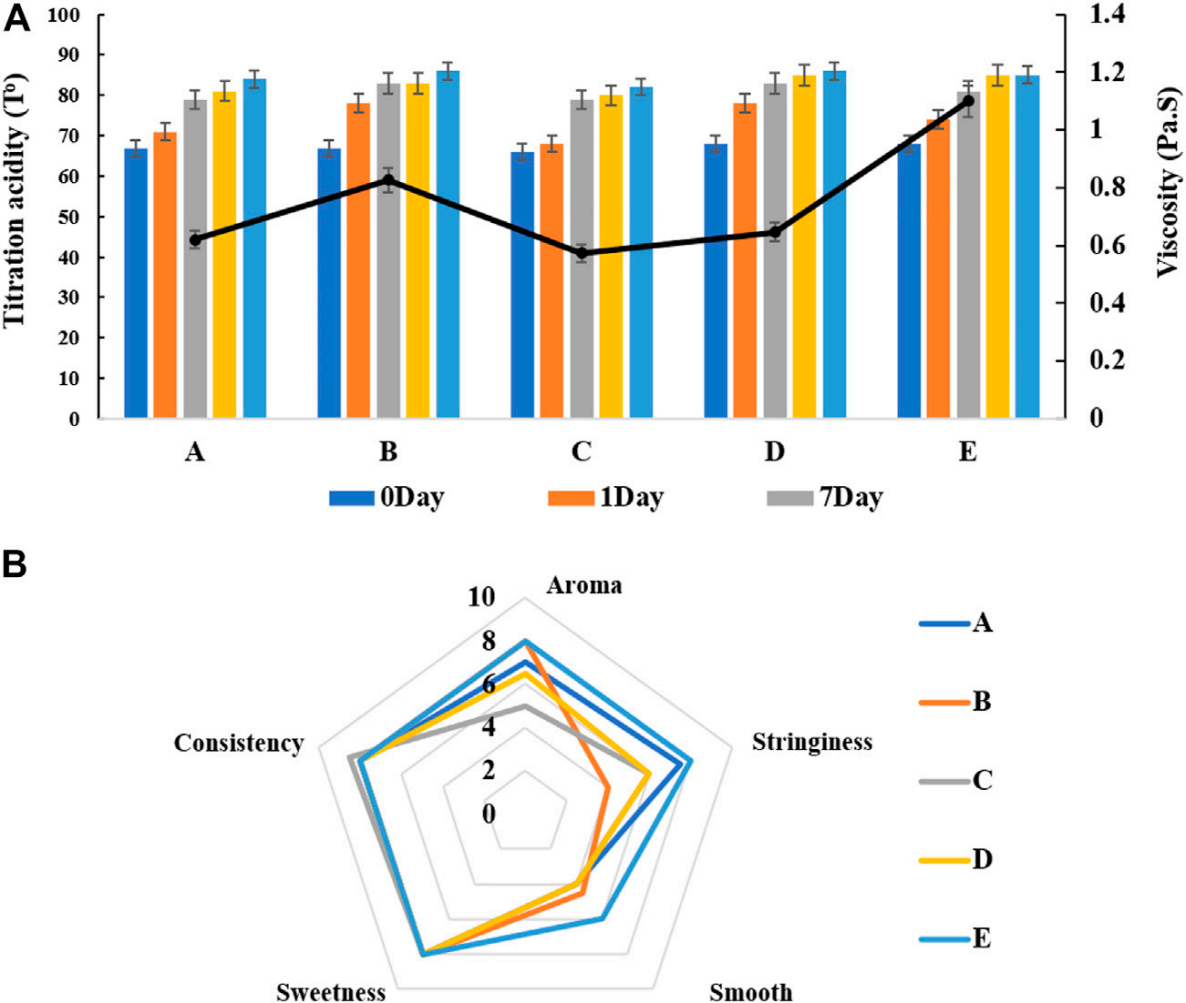
The viscosity, post-acidification **(A)** and sensory evaluation **(B)** of fermented milk produced by different mixed strains compounds. A ST53:ST49:ST50 = 1:1:1 B ST53:ST49:ST50 = 1:3:2 C ST53:ST49:ST50 = 3:1:2 D ST53:ST49:ST50 = 2:2:1 E ST53:ST49:ST50 = 1:2:2.

Our mixed fermentation experiments showed that ST53, ST49, and ST50 promoted one another’s growth (Figure 6), and that post-acidification by the ST53 + ST50 and ST53 + ST49 co-cultures was lower than that by the ST49 + ST50 co-culture; thus, we further studied the characteristics of co-culture strategies using these strain combinations. The viscosity, post-acidification, and sensory evaluation results of the yogurt samples produced using various co-cultures were shown in Figure 7. Compound E yielded the highest viscosity, the second lowest post-acidification, and the best flavor. Thus, a DVS starter culture comprised of ST53 + ST49 + ST50 at a ratio of 1:2:2 was selected to explore its post-acidification capability and resulting yogurt quality.

### Rheological properties of the fermented yogurt

**FIGURE 8:**
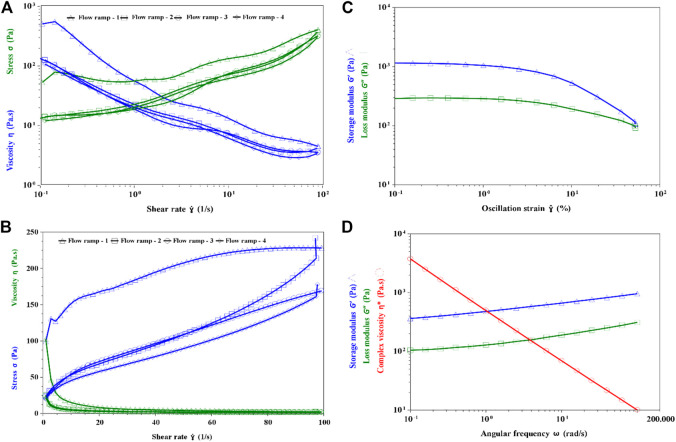
Rheological properties of the fermented yogurt. **(A)** Flow ramp of yogurt in linear **(B)** Flow ramp of yogurt in Log **(C)** Linear viscoelastic region test **(D)** Oscillation frequency test.

The rheological properties of the fermented yogurt produced by Compound E was studied. The apparent viscosity decreased with the increase in the shearing rate, but the sample had good ability to withstand mechanical handling (Fig. 8AB). When the shearing rate increased or decreased from 0.1/s to 100/s, the viscosity of the stirred yogurt samples remained nearly the same from the second flow ramp. This indicated that the yogurt produced by Compounds E had the ability to withstand mechanical handling during dairy processing and transport. The thixotropic properties of fresh yogurt have been well established in previous studies (Sakin-Yilmazer et al., 2014; Xu et al., 2018). These could be quantified by the yogurt’s ability to regain its initial structure when allowed to rest for a certain period after disturbing.

The linear viscoelastic region γ < 1% (Figure 8C), γ = 0.5% was selected for the oscillation test. From 0.1 to 100 rad/s, the G′ of yogurt sample remained larger than G″, indicating that the structure of EPS and protein networks had higher elasticity than viscosity (Figure 8D). A higher elasticity of yogurt is known to reduce its stringiness.

## Conclusion

The acidification rate and EPS production by *S. thermophilus* strains influence the gel formation and final texture of fermented yogurt. In this study, the phenotypic characteristics such as the acidification rate, viscosity, and different carbon source fermentation ability of 100 *S. thermophilus* strains were investigated, as well as the post-acidification capacity of monocultures and co-cultures of these strains. A co-culture strategy that yielded yogurt with high viscosity, low post-acidification, and good sensory properties was identified. The rheological properties revealed that samples fermented by co-cultures had a good ability to withstand mechanical handling during dairy processing and transport.

## Data Availability

The original contributions presented in the study are included in the article/Supplementary Material, further inquiries can be directed to the corresponding author.
